# Supplementary material for: Gene drives as a new quality in GMO releases—a comparative technology characterization
Source: PeerJ. 2019 May 3;7:e6793. doi: 10.7717/peerj.6793 (PMC6501761; doi:10.7717/peerj.6793)
Supplement: Supplemental Information 1 — Information and images explaining the model. [file peerj-07-6793-s001.zip › Supporting information.docx]

## **Supporting information**

### **Explanation of the deterministic assessment of gene drive allele frequency**

To better understand the model, it will be explained on the example of Medea. In a Medea drive, there are only three different genotypes: Wildtype (+/+), heterozygous Medea carriers (M/+) and homozygous Medea carriers (M/M). A female of any of the three genotypes can potentially mate with a male of each of the three genotypes. This will produce offspring of those three genotypes with a certain probability. For instance, the probability that a mating of a wildtype female with a wild type male will produce wild type offspring is 1, while the probabilities for heterozygous or homozygous Medea offspring are 0. If a wild type female mates with a heterozygous Medea male (M/+) the probabilities are 0.5 for wild type offspring and 0.5 for heterozygous Medea offspring, while no homozygous Medea carriers can be sired. Note, that for each mate the cumulated probability for offspring regardless of genotype amounts to 1. (S1 Fig ).

**S1 Fig:** Punnett square for wild type females (+/+♀).
Male mates depicted in orange, offspring genotypes shown in grey. Numbers represent probabilities of the respective genotype in offspring.

However for wild type females, the Medea system does not deviate from the Mendelian laws of inheritance. This changes when focusing on the heterozygous Medea females. Here again, the female can potentially mate with males from any of the three genotypes. But if the offspring does not at least carry one Medea allele it will be non-viable. Therefore, if the female’s mate is wildtype or heterozygous for Medea, the cumulated probability for offspring is below 1. This is the point where the Medea technique deviates from the Mendelian Inheritance (S2 Fig).

**S2 Fig:** Punnett square for heterozygous Medea females (M/+♀).
Male mates depicted in orange, offspring genotypes shown in grey. Numbers represent probabilities of the respective genotype in offspring. Offspring without a Medea allele are non-viable (red).

Finally, the mother can also be a homozygous Medea carrier. In this case, regardless of the male’s genotype all offspring will inherit at least one Medea allele and thus be viable. Here again the cumulated probability of all mates to sire any offspring is 1 (S3 Fig).

**S3 Fig:** Punnett square for homozygous Medea females (M/M♀).
Male mates depicted in orange, offspring genotypes shown in grey. Numbers represent probabilities of the respective genotype in offspring. Mating with homozygous Medea carriers will never sire wildtype offspring.

In the next step, the mating probabilities have to be established. In this approach, it is assumed that mating is random and only dependent on the occurrence of a genotype in a population. It is more likely for a wildtype female to mate with a wild type male when the wild type population percentage is higher than that of any other genotype. Furthermore, the probabilities for the emergence of offspring of a certain genotype are adapted to that genotype’s fitness. For instance, the Medea^dah^-construct proposed by (37) confers a fitness penalty to Medea carriers of 17.4%. Therefore, the fitness of M/+ and M/M organisms could be assumed as 0.826, while the wild type is assumed with a fitness of 1. The probabilities of the offspring genotypes in the Punnett squares is then multiplied with the respective fitness. This would change the probabilities of the offspring genotypes as depicted in S4 Fig.

**S4 Fig:** The previously shown Punnett squares now with the probabilities multiplied by the respective genotypes’ fitness.
The fitness for wild types is assumed to be 1, while hetero- and homozygous Medea carriers are assumed to have a fitness of 0.826, with respect to (37).

The last very important parameter which has to be chosen is the initial population percentage for each of the considered genotypes. This also determines how many gene drive organisms will be released. The approach assumes a single release. As an example, let’s assume a release of only homozygous Medea carriers. We release as many of them that 25% of the total population will be of M/M and 75% of wildtype genotype. 0% will be of M/+ genotype.

In the approach, each Punnett square is treated as a 3×3 matrix, at least for Medea (Equation 1), while the population percentages are treated as a 3 dimensional vector (Equation 2). This vector is multiplied with each of the three matrices, resulting in 3 vectors (Equation 3). These vectors multiplied with the respective population percentage and normalized by the sum of the vectors multiplied by the respective population percentage yield another 3 dimensional vector (Equation 4). This vector represents the population percentages of the subsequent generation. This process is then repeated with the newly yielded vector for the next generation and so on and so forth. This allows to monitor the development of a population over an indefinite number of generations.

$Punnett Square+/+♀=$***A*** =$\left| \begin{matrix} a_{11} & a_{12} & a_{13} \\ a_{21} & a_{22} & a_{23} \\ a_{31} & a_{32} & a_{33} \end{matrix} \right|$; (1)

$Punnett Square+/M♀=$***B*** =$\left| \begin{matrix} b_{11} & b_{12} & b_{13} \\ b_{21} & b_{22} & b_{23} \\ b_{31} & b_{32} & b_{33} \end{matrix} \right|$;

$Punnett Square M/M♀=$***C*** =$\left| \begin{matrix} c_{11} & c_{12} & c_{13} \\ c_{21} & c_{22} & c_{23} \\ c_{31} & c_{32} & c_{33} \end{matrix} \right|$;

$Population Percentage=\vec{{Pop}_{i}}=\begin{matrix} d_{11} & d_{12} & d_{13} \end{matrix}$ (2)

$\boldsymbol{A}\times\vec{{Pop}_{i}}=\vec{A}_{i}$; (3)

$$\boldsymbol{B}\times\vec{{Pop}_{i}}=\vec{B}_{i};$$

$$\boldsymbol{C}\times\vec{{Pop}_{i}}=\vec{C}_{i}$$

$Population Percentage of the next Generation= \vec{Pop}_{i+1}=\frac{\vec{A}_{i}\times d_{11}+\vec{B}_{i}\times d_{12}+\vec{C}_{i}\times d_{13}}{\sum{(\vec{A}}_{i}\times d_{11}+\vec{B}_{i}\times d_{12}+\vec{C}_{i}\times d_{13})}$ (4)

Following the example of an initial release of homozygous Medea-carriers with a fitness of 0.826, this results in a table of population percentages for the three genotypes over generations. This can be plotted and is illustrated in S5 Fig. The lines depict the population percentages of the wildtype (blue), the homozygous Medea-carriers (red) and the heterozygous Medea-carriers (grey). With the chosen fitness and initial population percentage the wild type will reach a 10^-4^, 24 generations post-release. The rest of the population consists of Medea-organisms. In the following generations, the percentage of heterozygotes declines while the percentage of homozygotes closes in on 100%.

**S5 Fig:** Population dynamics of a Medea gene drive with a fitness of 82.6% and an initial release percentage of 25% homozygotes.
Wildtype (blue), homozygous Medea Carriers (red), heterozygous Medea Carriers (grey). First the percentage of heterozygotes strongly increases until generation 20, while the wild type population percentage gradually drops with an increasing rate and the initially small percentage of homozygotes rises at increasing rate.

To complete the example, we now decrease the initial release percentage from 25% to 23%. Here we see, that with an initial release just lowered by 2%, there is no population replacement. The necessary invasion threshold is not reached and the genetically engineered organisms disappear from the hypothetical population over many generations (S6 Fig).

**S6 Fig:** Population dynamics of a Medea gene drive with a fitness of 82.6% and an initial release percentage of 23% homozygotes.
Wildtype (blue), homozygous Medea Carriers (red), heterozygous Medea Carriers (grey). Although at first the heterozygotes spike upwards in percentage and maintain this percentage around 30% after approximately 35 generations this subpopulation drops, and all gene drive organisms are eradicated from the population around 67 generations post-release.

To test this model’s validity, we again apply it to the results of (37). In caged trials, equal numbers of heterozygous Medea were mated with wildtypes. No more wildtypes were found after 12 to 16 generations. Therefore, in the next computation, we release heterozygotes with a release percentage of 50% (S7 Fig). Here, we see complete suppression of the wildtype genotype, the percentage is 3.9% at generation 12 and 0.02% at generation 16. This would not constitute a complete eradication in a large wild population of Drosophila. However, considering the population sizes in caged trials, the chance to find a wildtype would probably be slim. In conclusion, this far we may consider the model in accordance with the findings of caged trials.

**S7 Fig:** Population dynamics of a Medea gene drive with a fitness of 82.6% and an initial release percentage of 50% heterozygotes.
Wildtype (blue), homozygous Medea Carriers (red), heterozygous Medea carriers (grey).

### **Invasiveness**

In the model approach, whether a gene drive successfully replaces the wildtype population or is lost over the generations is majorly dependent on the two variable factors fitness and initial population percentage. These may vary with respect to the applied gene drive technique. Therefore, the approach seems suitable to prospectively quantify the invasiveness of gene drive techniques. Wherein, a gene drive technique that would achieve a complete population replacement within a certain amount of generations with a lower release threshold than another system would be deemed more invasive.

In this computational approach, only two major factors of such a gene drive system can be altered, namely fitness and initial population percentage. Although possible, it would be tedious to now iterate the variables by hand to determine the thresholds for a population replacement or a suppression of the wildtype, respectively. Instead, this is automated in a program written for this purpose in the following step. The program iterates each parameter in 1%-steps for a given generation post-release. This means the computation yields 10,000 data points (100 × 100), each a combination of fitness and release population percentage. The program judges upon variable thresholds in which out of three categories each of the data points is to be put. The three categories are: wildtype suppression, intermediate state and loss of gene drive construct. For most gene drive systems the thresholds were chosen as 5% and 95%. This means if the wildtype population percentage at a given post-release generation reached values below 5% the data point is considered in the suppression category. If the wildtype population percentage reached values above 95% the gene drive construct is considered lost for that data point. Any population percentages between 5% and 95% are considered as intermediate states.

Such a computation for a certain generation post-release can then be plotted in a cross section, illustrated in Fig S8. Here, the combinations of fitness and initial release population percentage of the Medea gene drive organisms after 10 generations post-release (pR) can be seen. Blue data points represent the disappearance of the gene drive organisms from the population. Red data points represent a suppression of the wildtype population and grey data points represent intermediate states in which wildtype and gene drive organisms coexist.

**S8 Fig:** Cross section of Medea gene drive release of homozygotes for the 10^th^ generation post-release.
(pR).Red = Wildtype population percentage below 5%; Blue = Wildtype population percentage above 95%; Grey = Wildtype population between 5% and 95%.

In order to evaluate the obtained data with respect to the invasiveness, two things are needed. First, this is just a freeze frame of the gene drives characteristics. To be able to draw rational conclusions its development over time has to be examined. Second, a comparison to other gene drive systems’ development over time has to be established.

In the following S9 Fig, the cross sections for Medea are shown. The depicted generations pR from left to right are 10, 15, 20 and 25. It can be observed that the area of the intermediate state is shrinking over time. The red and blue areas are growing slowly. It may be estimated that the intermediate state will completely disappear over time and that either the suppression of wildtypes or the disappearance of gene drive organisms will occupy the larger area. This seems to be a suitable set of criteria to evaluate the invasiveness of a gene drive technique.

**S9 Fig:** Cross sections for Medea gene drive release of homozygotes for the 10^th^ to 25^th^ generation pR.
Red = Wildtype population percentage below 5%; Blue = Wildtype population percentage above 95%; Grey = Wildtype population between 5% and 95%. Grey area shrinks, blue and red areas grow over time.

Finally, to be able to more easily track the growth of the areas, the graphics shown above are displayed in a transparent overlay in S10 Fig. This image shows every fifth generation post release up to 45 generations pR. For clarity, black lines were added manually to discern the different examined generations but are omitted when these lines would have been too close to each other. Furthermore the intermediate states are omitted. It can be observed, that the blue area’s growth is higher than that of the red area. In both areas the growth declines with higher generations and is only marginally in the last generations.

**S10 Fig:** Cross section overlay of Medea gene drive release of homozygotes up to 45 generations post-release.
Red = Wildtype population percentage below 5%; Blue = Wildtype population percentage above 95%. Black numbers and lines represent the respective generation post-release.
