# Supplementary figures and images for: Gene drives as a new quality in GMO releases—a comparative technology characterization
Source: PeerJ. 2019 May 3;7:e6793. doi: 10.7717/peerj.6793 (PMC6501761; doi:10.7717/peerj.6793)

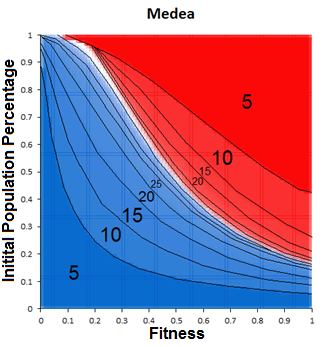

Supplement: Supplemental Information 1 — Information and images explaining the model. [file peerj-07-6793-s001.zip › S10 Fig.tif]

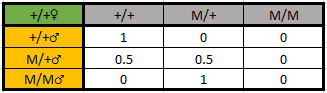

Supplement: Supplemental Information 1 — Information and images explaining the model. [file peerj-07-6793-s001.zip › S1 Fig.tif]

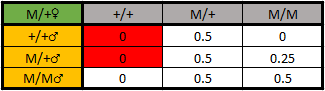

Supplement: Supplemental Information 1 — Information and images explaining the model. [file peerj-07-6793-s001.zip › S2 Fig.tif]

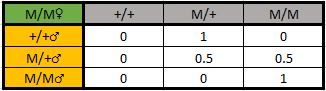

Supplement: Supplemental Information 1 — Information and images explaining the model. [file peerj-07-6793-s001.zip › S3 Fig.tif]

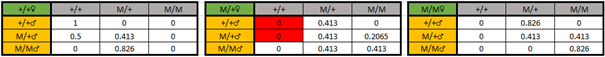

Supplement: Supplemental Information 1 — Information and images explaining the model. [file peerj-07-6793-s001.zip › S4 Fig.tif]

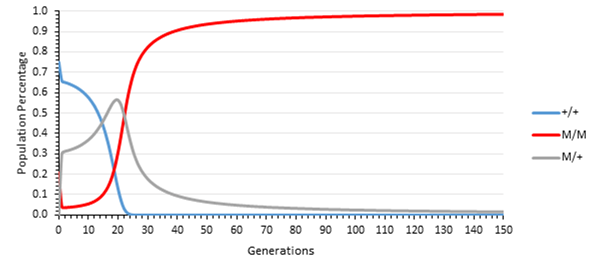

Supplement: Supplemental Information 1 — Information and images explaining the model. [file peerj-07-6793-s001.zip › S5 Fig.tif]

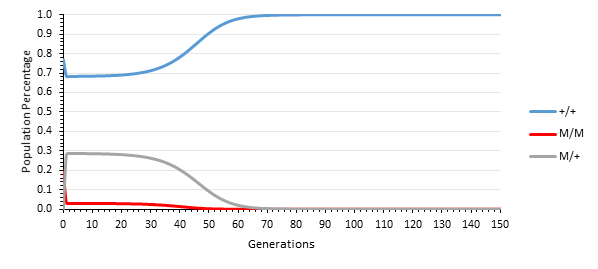

Supplement: Supplemental Information 1 — Information and images explaining the model. [file peerj-07-6793-s001.zip › S6 Fig.tif]

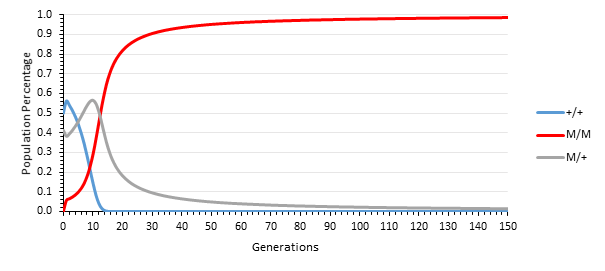

Supplement: Supplemental Information 1 — Information and images explaining the model. [file peerj-07-6793-s001.zip › S7 Fig.tif]

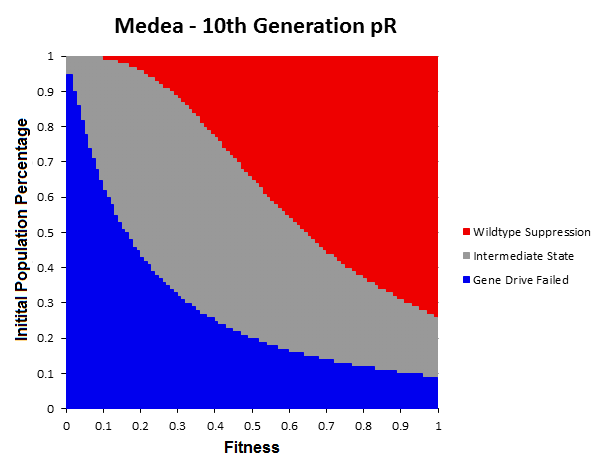

Supplement: Supplemental Information 1 — Information and images explaining the model. [file peerj-07-6793-s001.zip › S8 Fig.tif]

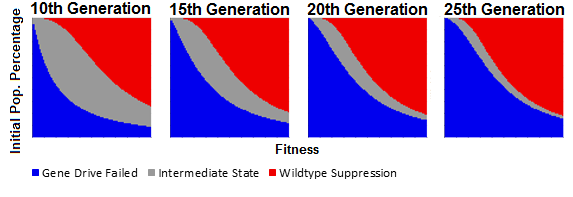

Supplement: Supplemental Information 1 — Information and images explaining the model. [file peerj-07-6793-s001.zip › S9 Fig.tif]

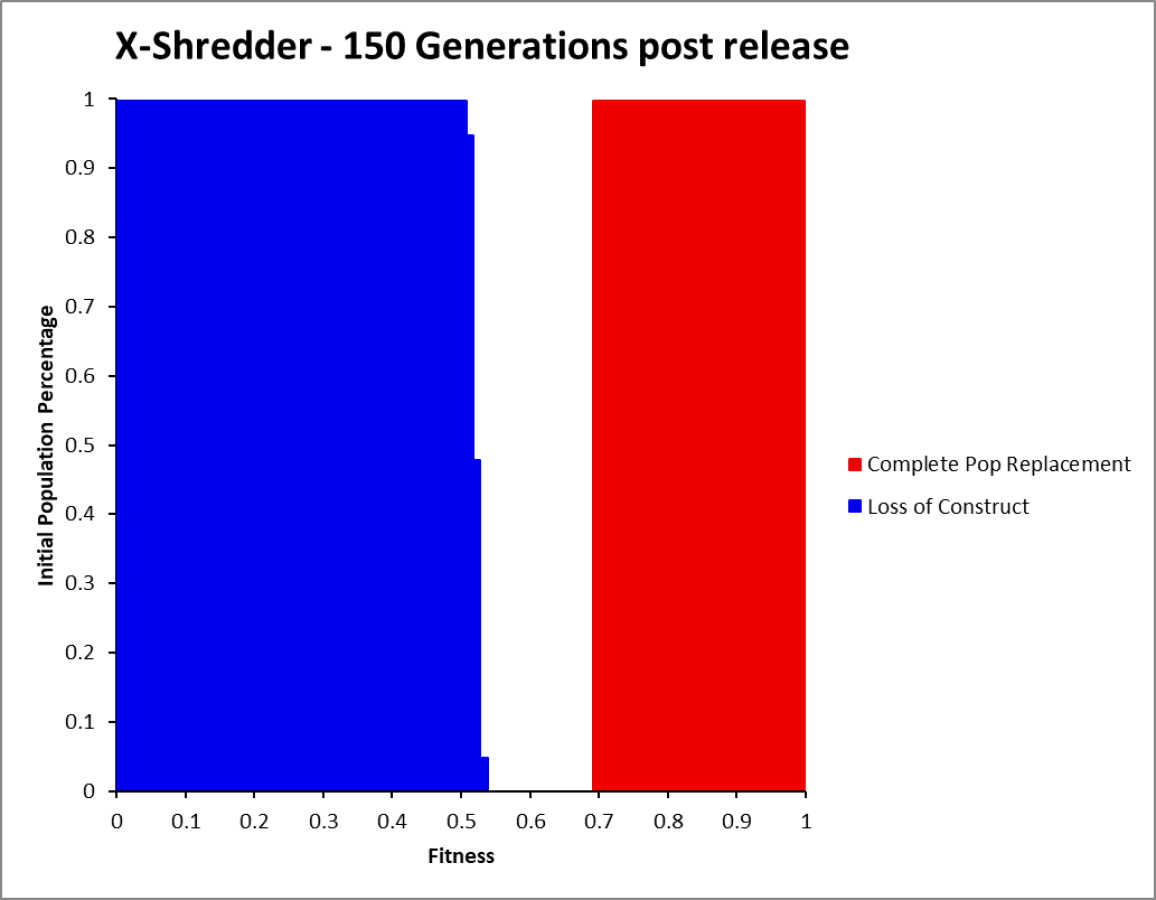

Supplement: Supplemental Information 1 — Information and images explaining the model. [file peerj-07-6793-s001.zip › S11 Fig-R1.png]
